# Supratentorial intraparenchymal schwannoma in a 44-year-old woman: A rare case report

**Published:** 2018-10-07

**Authors:** Mehdi Khaleghi, Alireza Arefidoust, Rahele Yaftian, Omid Ghamarnejad, Seyyed Mohammad Ghodsi

**Affiliations:** 1 Department of Neurosurgery, Shariati Hospital, Tehran University of Medical Sciences, Tehran, Iran; 2 Cerrahpaşa Faculty of Medicine, Istanbul University, Istanbul, Turkey

**Keywords:** Supratentorial, Schwannoma, Case Report

Intracerebral schwannoma has been known as a rare entity of intracranial tumors. It has been demonstrated that Schwann cells commonly arise from the peripheral nervous system, and do not exist in the parenchyma of the brain. The etiology and pathogenesis of these tumors in this uncommon location is unclear. However, several hypotheses have been concerned on the development and pathogenesis of these tumors. One of them supposed that schwannomas may originate from small vessels in the cortical and periventricular area, but Feigin and Ogata have suggested that mesenchymal multipotential cells may differentiate into Schwann cells.^1^ Srinivas, et al. categorized histogenesis theories into non-developmental and developmental. Non-developmental theories suggest that these tumors originate from Schwann cells, which display in the perivascular neural plexuses. The developmental theories involve the transformation of pial cells to Schwann cells, multipotential mesenchymal elements differentiation to Schwann cells, distorted embryogenesis, misplaced myelinated nerve ﬁbers acting as an origin site for tumor development, and translocated neural crest cells.^2^

In this study, we report a 44-year-old, right-handed woman, who complained of a severe bifrontal headache from one year that followed by binocular diplopia for one month with the experience of intermittent nausea and vomiting during last year. She had no family history of neurological disorders or malignancy. Upon admission, in the examinations, she had bilateral grade 2 papilledema and mild paresis of the right 6^th^ nerve. The cognition and other neurological exams were normal. She had no usual neurofibromatosis skin signs. She was evaluated with a brain computed tomography (CT) scan that disclosed a well-defined globular isodense space-occupying lesion, which was measured about 4 × 2.5 cm in the right frontal lobe. Additionally, a significant peritumoral edema was revealed. Moreover, magnetic resonance imaging (MRI) was performed, which displayed an isointense well-defined solid extra-axial mass on T1-weighted images involving the right frontal lobe (Figure 1, A). The lesion had slightly hyperintense-signal intensity on T2-weighted images, as well as had remarkable enhancement after gadolinium injection. 

**Figure 1:**
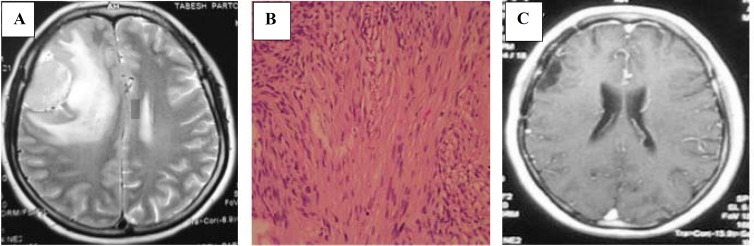
A: Axial T2-weighted magnetic resonance imaging (MRI) sequence showing a well-defined homogenous and slightly hyperintense mass with significant peritumoral edema and midline shift in the right frontal lobe. B: The tumor histopathological features, hematoxylin and eosin staining (×100) of the specimens reveals hypercellular and hypocellular regions. The hypercellular regions contain interlacing fascicles of elongated spindle cells with wavy nuclei that align in a palisading arrangement and formation of Verocay bodies in some regions (Antoni A). Other areas are hypocellular with myxoid background (Antoni B)

C: Axial T1-weighted MRI with contrast enhancement, about 3 months after operation, showed total tumor resection without evidences of tumor recurrence.

Based on these findings, the preoperative diagnosis was meningioma and afterward, a surgical approach was planned.

The patient underwent right frontolateral craniotomy, and gross total resection was achieved. Thereafter, hematoxylin and eosin staining was applied. Histological analysis of the specimens was characteristic for schwannoma (Figure 1, B). Immunohistochemistry investigations manifested that the cells of the tumor were generally reactive for S-100 protein and in the other hand, displayed a patchy positivity for glial fibrillary acid protein (GFAP) in a multifocal distribution, but negative for epithelial membrane antigen (EMA).

After the operation, the general situation of the patient was well, and she had no new neurological deficit. She was discharged from our hospital four days after the surgery. After three months, on follow-up examination, physical exams were normal, and she did not complain about any neurological problems. Furthermore, at the time of follow-up, MRI with and without Gadolinium showed no evidence of residual or recurrent tumor (Figure 1, C).

Ultimately, it has been suggested that, supratentorial intraparenchymal schwannomas are so rare in the brain, and also these tumors are extremely uncommon in the patients older than 30 years old, and have male predominance,^3^ but neurosurgeons should consider this entity as a possible differential diagnosis of a young and even elderly patients with an intracerebral space occupying lesion. The current case is supporting this theory that intracerebral schwannoma may be a developmental tumor. Intraparenchymal schwannomas look like cystic-solid on MRI,^4^ but it is not a characteristic appearance, and may be mistaken by the other tumors such as meningiomas. The pathology of intracerebral schwannoma is usually benign, and patients with total tumor resection have favorable outcomes.^3^ In the histological study, they have a different admixture of compact spindle cell area (Antoni A), as well as hypocellular and myxoid area with microcytic change (Antoni B) rich in fibers of collagen and macrophages. By immunohistochemistry study, schwannomas generally present strong and spread S100 protein expression. Moreover, GFAP can be positive in some subgroups of schwannomas.^5^ Schwannomas, as our case, are typically negative for EMA. On the other hand, fibroblastic meningioma tumors are moderately reactive to S-100, and EMA is generally positive. Accordingly, meningioma could exclude from the diagnosis.^3^

For better management of patients with supratentorial intraparenchymal schwannomas, it is worthy of further study about these tumors.

## Notes:

**How to cite this article:** Khaleghi M, Arefidoust A, Yaftian R, Ghamarnejad O, Ghodsi SM. Supratentorial intraparenchymal schwannoma in a 44-year-old woman: A rare case report. Iran J Neurol 2018; 17(4): 200-2.
